# Metabolic Syndrome Is Associated with Cataract in a Large Taiwanese Population Study

**DOI:** 10.3390/nu14091684

**Published:** 2022-04-19

**Authors:** Jung-Hsiu Chang, I-Hua Chen, Jiun-Hung Geng, Pei-Yu Wu, Jiun-Chi Huang, Szu-Chia Chen

**Affiliations:** 1 Department of Post Baccalaureate Medicine, Kaohsiung Medical University, Kaohsiung 807, Taiwan; jasonjrchang@gmail.com; 2 Division of Endocrinology and Metabolism, Department of Internal Medicine, Kaohsiung Medical University Hospital, Kaohsiung Medical University, Kaohsiung 807, Taiwan; control521@gmail.com; 3 Department of Urology, Kaohsiung Municipal Siaogang Hospital, Kaohsiung Medical University, Kaohsiung 812, Taiwan; u9001090@gmail.com; 4 Department of Urology, Kaohsiung Medical University Hospital, Kaohsiung Medical University, Kaohsiung 807, Taiwan; 5 Department of Internal Medicine, Kaohsiung Municipal Siaogang Hospital, Kaohsiung Medical University, Kaohsiung 812, Taiwan; wpuw17@gmail.com (P.-Y.W.); karajan77@gmail.com (J.-C.H.); 6 Division of Nephrology, Department of Internal Medicine, Kaohsiung Medical University Hospital, Kaohsiung Medical University, Kaohsiung 807, Taiwan; 7 Faculty of Medicine, College of Medicine, Kaohsiung Medical University, Kaohsiung 807, Taiwan; 8 Research Center for Environmental Medicine, Kaohsiung Medical University, Kaohsiung 807, Taiwan

**Keywords:** metabolic syndrome, cataract, Taiwan Biobank

## Abstract

Cataract is the leading cause of blindness worldwide, and metabolic syndrome (MetS) is a known risk factor. In this study, we investigated the association between the risk of cataract with MetS and its components in a large-scale study. Data were derived from the Taiwan Biobank, and 121,380 individuals were included. The NCEP-ATP III criteria modified for use in an Asian population were used to define MetS and its components. The occurrence of cataract was identified through a standardized interview and self-reported questionnaire. Multivariable analysis showed that MetS (OR, 1.129; 95% CI, 1.0175–1.184; *p* < 0.001), low high-density lipoprotein (HDL)-cholesterol (OR, 1.057; 95% CI, 1.005–1.113; *p* = 0.032), and hyperglycemia (OR, 1.162; 95% CI, 1.108–1.218; *p* < 0.001) were significantly associated with cataract. Furthermore, a stepwise increase in the prevalence of cataract corresponding to the number of MetS components was found. The presence of three MetS components (vs. 0; OR, 1.103; 95% CI, 1.024–1.188; *p* = 0.010), four MetS components (vs. 0; OR, 1.137; 95% CI, 1.040–1.242; *p* = 0.005), and five MetS components (vs. 0; OR, 1.208; 95% CI, 1.059–1.378; *p* = 0.005) were significantly associated with cataract. In conclusion, significant associations were found between a high incidence of cataract with MetS and its components, including low HDL-cholesterolemia and hyperglycemia. Further, a stepwise increase in the prevalence of cataract corresponding to the number of MetS components was also found. The results of this study indicate that MetS may increase the development of cataract in Taiwan.

## 1. Introduction

Cataract is defined as opacity of the crystalline lens, and approximately 12% of adults aged >40 years in Taiwan were reported to have cataract in 2013 [1]. Age, smoking [2,3], alcohol use [3], sunlight exposure [4], metabolic syndrome (MetS) [5], diabetes mellitus (DM) [3,5], and systemic corticosteroid use [6] are known risk factors for cataract, as they may induce either damage to the lens or effect the water–electrolyte balance of the aqueous humor [7,8], which will then change the transparency of the crystalline lens. Cataract has been associated with a poor quality of life and psychosocial distress [9,10], and typical symptoms include glare, diplopia, and visual dysfunction. Moreover, cataract is the leading cause of blindness worldwide [11], especially in low- and middle-income regions, where it has been reported to account for one in six cases of visual dysfunction and one in three cases of blindness [12]. Therefore, it is very important to detect the risk factors as early as possible in high-risk populations to allow for both prompt interventions and education on how to prevent cataract. The relationship between exercise and cataracts is another interesting topic due to the need to establish some preventive strategies. Jiang et al. [13] performed a dose-response meta-analysis to evaluate the relation between physical activity and age-related cataract (ARC) risk and revealed in quantity that the risk of ARC decreased by 2% for every 6 metabolic equivalents of task per day increase in activity. In addition, according to a Cox proportional hazard analyses conducted by Williams et al. [14], which assessed the different effects on cataract risk decrease between vigorous (running) and moderate exercise (walking), the results showed both were significantly associated with lower cataract risk and increasing exercise energy expenditure was associated with decrease of cataract risk linearly.

MetS is a cluster of metabolic abnormalities that include hyperlipidemia, hypertension, obesity, and insulin resistance [15,16,17]. However, the definition of MetS varies between guidelines such as the International Diabetes Federation (IDF), World Health Organization, Adult Treatment Panel III (ATP III), and others. Overall MetS prevalence rates of 15.7% and 22% have been reported in Taiwan and the United States, respectively [18,19]. In addition, MetS has been significantly associated with type 2 DM [20] and cardiovascular disease [21], and previous studies and animal experiments have revealed that MetS and its components may be biochemically and genetically related to cataract [22,23,24]. Different mechanisms have been postulated for the pathophysiological pathways between MetS and cataract [25,26], for example, lens protein glycation induced by DM leading to an increase in the hyperosmotic effects of sorbitol [24], and obesity-associated hyperleptinemia and leptin resistance resulting in the accumulation of reactive oxygen species, which has been strongly linked to lens opacification [27,28]. However, few large-scale studies have investigated the association between MetS and its components with cataract [22,29,30].

Four previous studies have reported on associations between cataract and/or cataract extraction with MetS [5,22,31,32]. Lindblad et al. examined the associations among middle-aged and older women and men [5,32], and Ghaem et al. reported that high glucose and obesity were associated with a higher incidence of cortical cataract over a 5 year period [22].

Due to the lack of studies using the ATP III criteria for MetS, we conducted this study using data from the Taiwan Biobank (TWB) on over 120,000 Taiwanese participants to evaluate the relationships among the five components of MetS defined according to the ATP III criteria and cataract. In addition, we also analyzed relationships between the number of MetS components and cataract. The aim of this study was to evaluate whether MetS, its components, and the number of MetS components were associated with a higher risk of cataract.

## 2. Materials and Methods

### 2.1. Taiwan Biobank

The TWB was established by the Ministry of Health and Welfare and approved by the Institutional Review Board on Biomedical Science Research, Academia Sinica, Taiwan, and the Ethics and Governance Council of the TWB. It includes data on community-dwelling people aged between 30 and 70 years with no prior history of cancer including lifestyle, genetic and medical factors [33,34]. All enrollees in the TWB undergo interviews and physical examinations, and blood samples are obtained. In this study, we included 121,390 participants, all of whom provided written informed consent, and the study was approved by the Institutional Review Board of Kaohsiung Medical University Hospital (KMUHIRB-E(I)-20210058) on 8 April 2021. In addition, this study followed the Declaration of Helsinki [35].

### 2.2. Collection of Study Variables

Hip circumference (HC), body height (BH), waist circumference (WC), body weight (BW), systolic blood pressure (BP) and diastolic BP were recorded during physical examinations. HC, BH, WC, and BW were measured once. BP data were measured by trained personnel by using a digital BP machine. The patients were advised to avoid caffeine, exercise, and smoking for at least 30 min before the first BP measurement. Each BP was measured three times, and separate repeated measurements were done in 1–2 min intervals. We used the average value of three readings of systolic BP and diastolic BP for later analysis. All participants enrolled in the TWB complete a questionnaire during an in-person interview. Data on personal medical history (such as hypertension and DM), lifestyle factors (such as exercise), sex and age were obtained using a questionnaire during the interviews. For the purpose of this study, we defined regular exercise as participating in an activity such as a sport, jogging, swimming, yoga, cycling, hiking, and exercise-based apps, for more than 30 min on three or more occasions in one week. Activities related to employment were not included in this study.

The following variables were also recorded: estimated glomerular filtration rate (eGFR—calculated using the Modification of Diet in Renal Disease study equation [36]), total cholesterol, low-density lipoprotein cholesterol (LDL-C), high-density lipoprotein cholesterol (HDL-C), triglycerides, fasting glucose, hemoglobin, and uric acid.

### 2.3. Definitions of Cataract and MetS

The participants were also asked if they had ever had cataract, and those who answered “Yes” were classified into the cataract group.

MetS was defined as the presence of three or more of the following criteria in accordance with the modified NCEP-ATP III criteria for use in an Asian population [37]: (1) systolic/diastolic BP of ≥130 mmHg or ≥85 mmHg, respectively, participants with a diagnosis of hypertension, and those prescribed hypertensive medications; (2) triglyceride concentration ≥150 mg/dL; (3) HDL-cholesterol concentration in women and men of <50 mg/dL and <40 mg/dL, respectively; (4) abdominal obesity (WC in women and men of ≥80 cm and ≥90 cm, respectively); (5) hyperglycemia (participants with a fasting whole-blood glucose concentration ≥100 mg/dL or a diagnosis of DM).

### 2.4. Statistical Analysis

Statistical Package for the Social Sciences version 19.0 was used to conduct the analysis (SPSS Inc., Chicago, IL, USA). Continuous variables are given as means ± standard deviations, and compared using the independent t test. Categorical variables are given as frequencies and percentages, and compared using the chi-squared test. Associations among MetS, the number of MetS components, and individual components with cataract were analyzed with multivariable logistic regression analysis, which included significant variables in univariable analysis. A *p*-value less than 0.05 was considered statistically significant.

## 3. Results

A total of 121,380 participants (mean age 49.9 ± 11.0 years; 43,616 men; 77,764 women) were enrolled in this study. All participants were classified into cataract (*n* = 17,320; 9.0%) and non-cataract (*n* = 110,413; 91.0%) groups.

### 3.1. Comparisons of the Characteristics between the Cataract and Non-Cataract Groups

Comparisons of the characteristics between the two groups are shown in Table 1. Compared to the non-cataract group, the cataract group were predominantly female, older, had higher prevalence rates of DM and hypertension, and lower prevalence rates of smoking and alcohol history. They also had a higher rate of regular exercise, higher systolic BP, diastolic BP, WC, fasting glucose, triglycerides, total cholesterol, and HDL-C, and lower HC, BH, BW, hemoglobin, and eGFR. Regarding MetS, the participants with cataract had a high prevalence of MetS, higher number of MetS components, and higher prevalence of all five components (central obesity, low HDL-C, hypertension, hypertriglyceridemia, and hyperglycemia).

### 3.2. Association of MetS and Cataract

Multivariable logistic regression analysis was conducted (adjusted for eGFR, uric acid LDL-C, total cholesterol, hemoglobin, age, sex, smoking habit, alcohol consumption, and regular exercise) to identify the factors associated with cataract, and the results are shown in Table 2. The results showed that MetS (odds ratio [OR], 1.129; 95% confidence interval [CI], 1.0175–1.184; *p* < 0.001), low uric acid (*p* < 0.001), low eGFR (*p* = 0.008), low LDL-C (*p* < 0.001), low hemoglobin (*p* < 0.001), smoking history (*p* < 0.001), female sex (*p* < 0.001), and older age (*p* < 0.001) were significantly associated with cataract.

### 3.3. Associations between the Number of MetS Components and Cataract

Associations between the number of MetS components and cataract were analyzed using multivariable logistic regression analysis adjusted for eGFR, uric acid LDL-C, total cholesterol, hemoglobin, age, sex, smoking habit, alcohol consumption, and regular exercise (Table 3). The results showed that the participants with three MetS components (vs. 0; OR, 1.103; 95% CI, 1.024–1.188; *p* = 0.010), four MetS components (vs. 0; OR, 1.137; 95% CI, 1.040–1.242; *p* = 0.005), and five MetS components (vs. 0; OR, 1.208; 95% CI, 1.059–1.378; *p* = 0.005) were significantly associated with cataract.

### 3.4. Associations between MetS Components and Cataract

Associations between the MetS components and cataract were analyzed using multivariable logistic regression adjusted for uric acid, eGFR, LDL-C, HDL-C, total cholesterol, triglycerides, hemoglobin, fasting glucose, diastolic BP, systolic BP, regular exercise, alcohol consumption, smoking history, hypertension, DM, sex, and age (Table 4). The results showed that the participants with low HDL-C (OR, 1.057; 95% CI, 1.005–1.113; *p* = 0.032) and hyperglycemia (OR, 1.162; 95% CI, 1.108–1.218; *p* < 0.001) were significantly associated with cataract. However, abdominal obesity (*p* = 0.806), hypertriglyceridemia (*p* = 0.094), and hypertension (*p* = 0.102) were not associated with cataract.

## 4. Discussion

The results of this large-scale study showed that after adjusting for confounding variables, the participants with MetS had a higher prevalence of cataract than those without MetS. In addition, in analysis of MetS components, low HDL-cholesterolemia and hyperglycemia were associated with a higher risk of cataract. Furthermore, we found a stepwise increase in cataract prevalence corresponding to the number of MetS components, although the trend was not significant.

In a prospective cohort study of 4508 women in Sweden who underwent cataract extraction with 98 months of follow-up, Lindblad et al. [5] investigated associations between the incidence of cataract extraction with MetS and its components. Although they lacked data on HDL-cholesterol and triglycerides, they found a 68% higher risk of cataract extraction in women with the other three components of MetS (hypertension, abdominal adiposity, and hyperglycemia) compared to women without these components. In particular, those under 65 years of age with these components had an approximately 3-fold higher risk of cataract extraction [5]. In another prospective cohort study of 45,049 men, Lindblad et al. [32] found similar results, in that men under 65 years of age or younger with abdominal adiposity, hyperglycemia, and hypertension had a relative risk of 2.43-fold for cataract extraction. In addition, in a prospective cohort study conducted in Australia, Ghaem et al. [22] reported that MetS defined according to the IDF criteria and its components were associated with the incidence of cortical cataract and posterior subcapsular cataract (PSC) over a 5-year period. In addition, they found that hyperglycemia was associated with the 10- and 5-year incidence rates of PSC cataract and cortical cataract, and low HDL-C was associated with the 10-year incidence of cortical cataract. In another cohort study conducted by Tan et al. [31], baseline MetS was associated with higher risks of incident PSC, nuclear, and cortical cataracts. In addition, significant associations were found between high MetS index values and cortical cataracts, and a borderline non-significant association with PSC cataract was also found [31]. In a population-based study conducted in Singapore, Sabanayagam et al. [30] investigated 2794 adults aged 40 to 80 years, and found a significant association between cataract and MetS only when the patients had ≥4 MetS components. The association between cataract and MetS has been proposed to be through oxidative stress and inflammatory processes [38,39]. Several studies have reported associations between cataract with pro-inflammatory cytokines such as tumor necrosis factor-α (TNF-α), interleukin-6 (IL-6), and C-reactive protein (CRP) [40,41]. In addition, many studies have shown an association between each component of MetS and increased levels of CRP and cytokines [42,43,44]. In addition, a linear association between an elevated CRP level with a higher number of MetS components has also been reported [45]. Oxidative stress and inflammation may therefore partially explain the association between MetS and cataract.

Another main finding is the significant association between a low HDL-C level and cataract. Ghaem et al. did not find a significant difference in the 10-year cumulative incidence of cataract between participants with or without low HDL-cholesterol; however, they found an association between a low-HDL-C level and a higher 10-year incidence of cortical cataract [22]. In addition, both Tan et al. and Sabanayagam et al. found no significant associations between low HDL-C and the incidence of cataract or cataract surgery [31]. Low HDL-C has been associated with higher CRP levels, especially in women [42,45], and Schaumberg et al. suggested that a high CRP level may be a marker for one of the fundamental biological mechanisms causing cataract [40]. Although some animal studies have reported associations between low HDL-C with oxidative stress and inflammatory processes leading to cataract formation, more accurate pathophysiological mechanisms linking low HDL-cholesterol with age-related cataract remain unclear [46,47].

We also found that the participants with hyperglycemia had a higher incidence of cataract, which is consistent with other cohort studies [5,22,31,32], cross-sectional studies [29,30,48], and a case-control study [49]. Two main mechanisms may contribute to cataract: First, a hyperosmotic effect leading to the accumulation of sorbitol in the lens, and subsequently swelling and rupture of lens fibers [50]. Second, the higher level of nonenzymatic glycation of protein in people with DM leading to an increase in the production of advanced glycation end products (AGEs) in the lens [51]. In addition, AGEs can induce TNF-α, IL-1α, and IL-6, which suggests that inflammation may link DM and cataract [44].

In addition, we did not find a significant association between abdominal obesity, defined by WC, and cataract. Many studies have reported a significant association between obesity and cataract, however most of them used body mass index (BMI) to define obesity, and only one included Asian people [5,22,31,52,53,54,55,56,57,58,59,60,61,62]. Only two prospective cohort studies have used WC to define obesity, both of which reported no significant association between cataract and obesity [32,57]. Using WC as a measure of obesity may be more appropriate in an Asian population because Asians tend to have more body fat per BMI than Caucasians [63], which indicates a greater potential for the development of hypertension, DM, and dyslipidemia at a lower BMI. In addition, due to the clear link between obesity and cataract caused by chronic inflammation and inflammatory markers such as CRP, IL-6 and TNF-α, and urinary isoprostanes, it is apparent that obesity is a risk factor contributing to cataract [42,43,64]. Therefore, despite the inconsistent results between studies using BMI and WC as the definition of obesity, obesity remains a strong risk factor for cataract.

Moreover, we did not find a significant association between hypertriglyceridemia and cataract in this study, which is consistent with many previous studies [22,31]. Ridker et al. investigated relationships between incident cardiovascular events, MetS, and CRP in 14,719 healthy women, and found that each component of MetS contributed to CRP level [45]. However, Tan et al. and Ghaem et al. reported that hypertriglyceridemia was not significantly related with incident cataract [22,31].

Finally, we found that high BP was not significantly associated with cataract. An association between cataract and hypertension has been reported in previous cohort studies [5,31,65], cross-sectional studies [30,48,66,67], and a case-control study [49], however the results were inconsistent. Associations between hypertension with TNF-α, IL-6 [68], and CRP [45] have been reported, suggesting that inflammation may link cataract and hypertension [40,41]. However, it was difficult to assess the degree of hypertension control with medications in each participant, and this may have confounded the analysis of the association between cataract and hypertension.

The main strengths of this study include the large cohort of healthy individuals living in the community, and that we controlled for confounding factors including smoking, lifestyle, and cardiovascular risk factors. However, there are also several limitations. First, data on the use of medications which may have influenced hypertension, the level of fasting glucose, lipid profiles, and the development or prevention of MetS such as anti-hypertensive, anti-diabetic, and lipid-lowering medications were not available in the TWB, and this may have led to an underestimation of the association between MetS and cataract. Second, due to the cross-sectional design of this study, we could not determine how long each participant had cataract. Therefore, we could not elucidate the causal relationship between cataract and MetS. Further longitudinal studies are warranted to investigate the risk of MetS and its components to incident cataract. Furthermore, we can further explore if intervention to improve MetS and its components can reduce the incidence of cataract. Third, the occurrence of cataract was determined through unverified self-reported questionnaires, and thus the type or severity of cataract could not be ascertained. Fourth, the enrolled participants were all ethnically Chinese, and this may limit the generalizability of our findings to other groups.

In conclusion, we found that MetS and its components, including low HDL-cholesterolemia and hyperglycemia, were associated with a high prevalence of cataract. Further, we found a stepwise increase in the prevalence of cataract corresponding to the number of MetS components. Our findings suggest that MetS may play a role in the risk of cataract in Taiwan.

## Figures and Tables

**Table 1:** Comparison of clinical characteristics among study participants according to cataract.

Characteristics	Cataract (−) (*n* = 110,413)	Cataract (+) (*n* = 10,967)	*p*
Age (year)	48.7 ± 10.6	61.3 ± 6.5	<0.001
Male gender (%)	36.2	32.8	<0.001
DM (%)	4.2	14.3	<0.001
Hypertension (%)	11.0	24.5	<0.001
Smoking history (%)	27.6	24.0	<0.001
Alcohol history (%)	8.6	7.9	0.021
Regular exercise habits (%)	38.7	59.2	<0.001
Systolic BP (mmHg)	119.8 ± 18.5	127.2 ± 19.1	<0.001
Diastolic BP (mmHg)	73.8 ± 11.4	74.3 ± 10.8	<0.001
Body height (cm)	162.2 ± 8.3	159.3 ± 7.9	<0.001
Body weight (kg)	64.0 ± 12.8	61.5 ± 11.3	<0.001
Waist circumference (cm)	83.2 ± 10.3	84.4 ± 9.9	<0.001
Hip circumference (cm)	96.1 ± 7.2	95.0 ± 6.7	<0.001
Laboratory parameters			
Fasting glucose (mg/dL)	95.3 ± 20.0	101.7 ± 26.3	<0.001
Hemoglobin (g/dL)	13.8 ± 1.6	13.7 ± 1.4	0.010
Triglyceride (mg/dL)	115.2 ± 94.6	119.8 ± 88.6	<0.001
Total cholesterol (mg/dL)	195.3 ± 35.8	198.7 ± 36.4	<0.001
HDL-C (mg/dL)	54.5 ± 13.4	55.0 ± 13.8	0.001
LDL-C (mg/dL)	120.9 ± 31.8	121.4 ± 31.8	0.120
eGFR (mL/min/1.73 m^2^)	104.0 ± 23.7	96.1 ± 24.0	<0.001
Uric acid (mg/dL)	5.4 ± 1.4	5.4 ± 1.4	0.058
MetS (%)	21.6	32.0	<0.001
MetS numbers	1.5 ± 1.3	1.9 ± 1.4	<0.001
MetS component			
Abdominal obesity (%)	45.8	52.9	<0.001
Hypertriglyceridemia (%)	20.8	22.6	<0.001
Low HDL-cholesterol (%)	25.4	26.9	0.001
Hyperglycemia (%)	19.5	33.4	<0.001
High blood pressure (%)	33.4	52.5	<0.001

Abbreviations. DM, diabetes mellitus; BP, blood pressure; HDL-C, high-density lipoprotein cholesterol; LDL-C, low-density lipoprotein cholesterol; eGFR, estimated glomerular filtration rate; MetS, metabolic syndrome.

**Table 2:** Association of MetS and cataract using multivariable logistic regression analysis.

Variables	Multivariable (Cataract)	
	Odds Ratio (95% CI)	*p*
Age (per 1 year)	1.173 (1.169–1.177)	<0.001
Male vs. female	0.766 (0.715–0.821)	<0.001
Smoking history	1.120 (1.051–1.193)	<0.001
Alcohol history	0.971 (0.892–1.055)	0.485
Regular exercise habits	1.043 (0.998–1.090)	0.061
Hemoglobin (per 1 g/dL)	0.957 (0.940–0.975)	<0.001
Total cholesterol (per 10 mg/dL)	1.009 (0.996–1.021)	0.176
LDL-cholesterol (per 1 mg/dL)	0.997 (0.996–0.998)	<0.001
eGFR (per 1 mL/min/1.73 m^2^)	0.999 (0.998–1.000)	0.008
Uric acid (per 1 mg/dL)	0.958 (0.940–0.977)	<0.001
MetS	1.129 (1.075–1.184)	<0.001

Values are expressed as odds ratio and 95% confidence interval (CI). Abbreviations. LDL-C, low-density lipoprotein cholesterol; eGFR, estimated glomerular filtration rate; MetS, metabolic syndrome.

**Table 3:** Association of MetS numbers and cataract using multivariable logistic regression analysis.

Number of MetS Components	Multivariable (Cataract)	
	Odds Ratio (95% CI)	*p*
0	Reference	
1	0.998 (0.935–1.066)	0.957
2	0.989 (0.924–1.059)	0.751
3	1.103 (1.024–1.188)	0.010
4	1.137 (1.040–1.242)	0.005
5	1.208 (1.059–1.378)	0.005

Values are expressed as odds ratio and 95% confidence interval (CI). Adjusted for eGFR, uric acid LDL-C, total cholesterol, hemoglobin, age, sex, smoking habit, alcohol consumption, and regular exercise.

**Table 4:** Association of MetS components and cataract using multivariable logistic regression analysis.

MetS Components	Multivariable (Cataract)	
	Odds Ratio (95% CI)	*p*
Abdominal obesity	1.006 (0.961–1.052)	0.806
Hypertriglyceridemia	1.047 (0.992–1.106)	0.094
Low HDL-cholesterol	1.057 (1.005–1.113)	0.032
Hyperglycemia	1.162 (1.108–1.218)	<0.001
High blood pressure	1.038 (0.993–1.086)	0.102

Values are expressed as odds ratio and 95% confidence interval (CI). Abbreviations. DM, diabetes mellitus; BP, blood pressure; HDL-C, high-density lipoprotein cholesterol; LDL-C, low-density lipoprotein cholesterol; eGFR, estimated glomerular filtration rate; MetS, metabolic syndrome. Adjusted for uric acid, eGFR, LDL-C, HDL-C, total cholesterol, triglycerides, hemoglobin, fasting glucose, diastolic BP, systolic BP, regular exercise, alcohol consumption, smoking history, hypertension, DM, sex, and age.

## Data Availability

The data underlying this study are from the Taiwan Biobank. Due to restrictions placed on the data by the Personal Information Protection Act of Taiwan, the minimal data set cannot be made publicly available. Data may be available upon request to interested researchers. Please send data requests to: Szu-Chia Chen, PhD, MD. Division of Nephrology, Department of Internal Medicine, Kaohsiung Medical University Hospital, Kaohsiung Medical University.

## Abbreviations

MetS	Metabolic Syndrome
DM	Diabetes Mellitus
IDF	International Diabetes Federation
ATP III	Adult Treatment Panel III
TWB	Taiwan Biobank
HC	Hip Circumference
BH	Body Height
WC	Waist Circumference
BW	Body Weight
BP	Blood Pressure
eGFR	Estimated Glomerular Filtration Rate
LDL-C	Low-Density Lipoprotein Cholesterol
HDL-C	High-Density Lipoprotein Cholesterol
OR	Odds Ratio
CI	Confidence Interval
PSC	Posterior Subcapsular Cataract
TNF-α	Tumor Necrosis Factor-α
IL-6	Interleukin-6
CRP	C-Reactive Protein
AGEs	Advanced Glycation End Products
BMI	Body Mass Index
